# High-throughput assay and engineering of self-cleaving ribozymes by sequencing

**DOI:** 10.1093/nar/gkv265

**Published:** 2015-03-30

**Authors:** Shungo Kobori, Yoko Nomura, Anh Miu, Yohei Yokobayashi

**Affiliations:** 1 Department of Biomedical Engineering, University of California, Davis, CA 95616, USA; 2 Nucleic Acid Chemistry and Engineering Unit, Okinawa Institute of Science and Technology Graduate University, Onna, Okinawa 904 0495, Japan

## Abstract

Self-cleaving ribozymes are found in all domains of life and are believed to play important roles in biology. Additionally, self-cleaving ribozymes have been the subject of extensive engineering efforts for applications in synthetic biology. These studies often involve laborious assays of multiple individual variants that are either designed rationally or discovered through selection or screening. However, these assays provide only a limited view of the large sequence space relevant to the ribozyme function. Here, we report a strategy that allows quantitative characterization of greater than 1000 ribozyme variants in a single experiment. We generated a library of predefined ribozyme variants that were converted to DNA and analyzed by high-throughput sequencing. By counting the number of cleaved and uncleaved reads of every variant in the library, we obtained a complete activity profile of the ribozyme pool which was used to both analyze and engineer allosteric ribozymes.

## INTRODUCTION

Recent advances in genome sequencing and bioinformatics have revealed the ubiquitous presence of self-cleaving ribozymes in all domains of life (1–4). The evidence that some natural RNAs can catalyze chemical reactions is one of the arguments supporting the RNA World hypothesis (5). As such, chemists have long been studying natural and artificial ribozymes with nucleolytic and other chemical activities. More recently, we and other groups have engineered allosteric ribozymes (aptazymes) by strategically fusing an RNA aptamer (6,7)—a molecular recognition RNA motif—with a self-cleaving ribozyme to chemically control gene expression in living cells (8,9). Regardless of the objectives, ribozyme studies often involve biochemical characterization of multiple individual ribozyme mutants, for example, to test hypotheses regarding the roles of specific nucleotides or to validate the activities of mutants obtained from screening or selection experiments. However, the number of variants that can be examined by conventional assays is severely limited because each ribozyme variant must be prepared and assayed individually.

**Figure 1.**
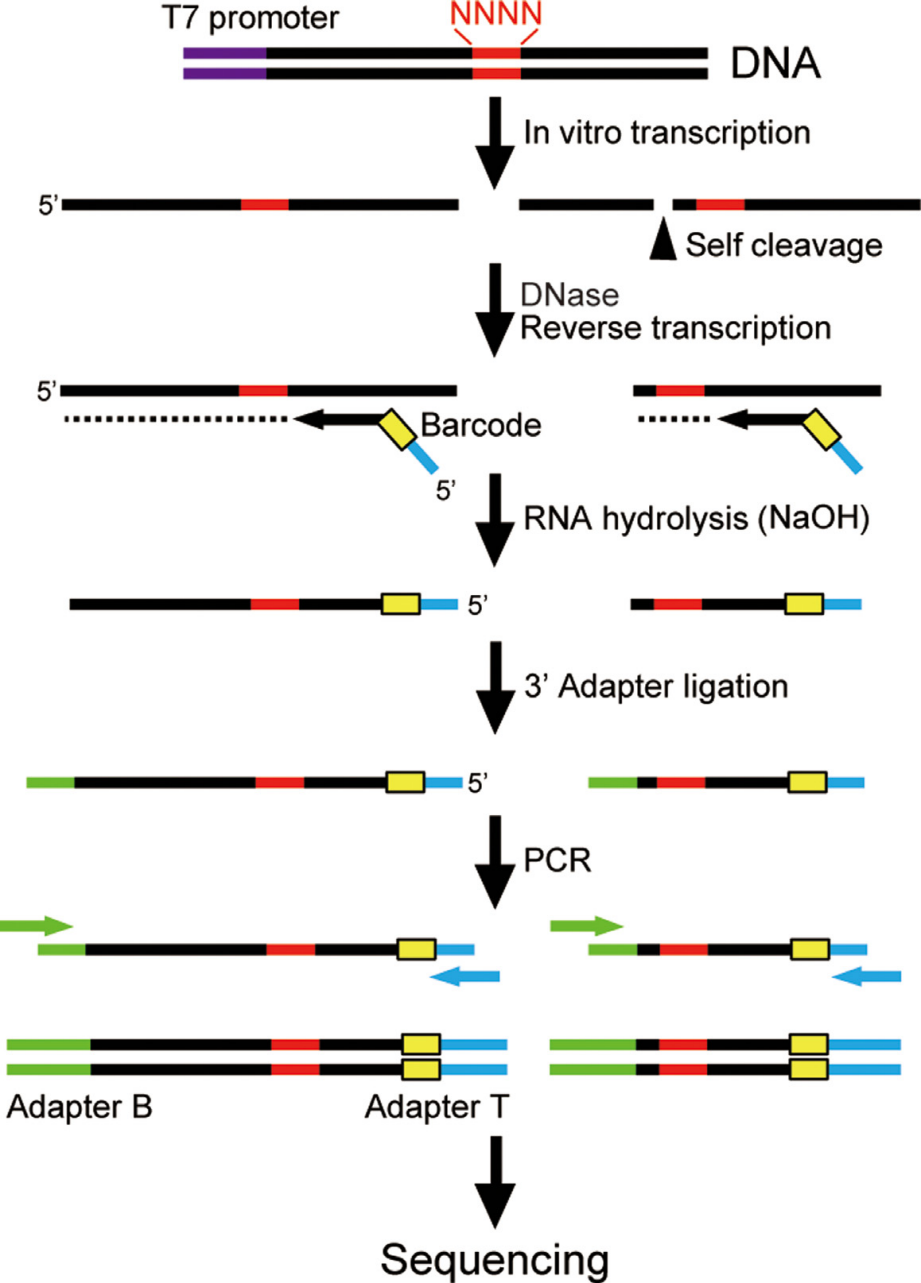
Library construction strategy. First, a partially randomized ribozyme library is transcribed *in vitro* from a DNA template. The cleaved and uncleaved ribozymes are reverse transcribed into cDNAs using a primer that contains a barcode and an adapter sequence. After removing the RNAs, the 3′ adapter is attached and amplified by PCR to obtain the sequencing library. The core ribozyme sequence is shown in black with the degenerate bases depicted in red. Other sequence elements: T7 promoter (purple), barcode (yellow), adapter sequences for sequencing (green and blue).

Here, we describe a simple strategy that allows quantitative assay of >1000 predefined ribozyme variants in parallel aided by high-throughput sequencing (HTS). The general approach is depicted in Figure 1. First, a library of ribozyme mutants is generated by *in vitro* transcription and allowed to undergo self-cleavage reaction under a desired condition. Second, the ribozyme library is converted to DNA and processed to attach adapter and barcode sequences necessary for HTS. At this stage, each DNA molecule carries the following information that originates from a single ribozyme molecule: the sequence of the varied bases, whether the ribozyme was cleaved or not, and the library and the reaction conditions encoded in the barcode. As in other HTS applications, barcoding allows one to run multiple experiments (e.g. different reaction conditions or ribozyme libraries) in a single sequencing session. Finally, the sequencing data are sorted to count the number of cleaved and uncleaved reads to assign a relative activity (fraction cleaved) to every variant in the library. It should be noted that this strategy has some important distinctions from conventional selection or screening of ribozyme libraries. Selection or screening typically identifies the sequences of a very small fraction of ‘winners’ in a large pool of variants that must be further characterized in detail as mentioned above. Our HTS ribozyme assay provides a complete sequence–activity profile of all variants in the library, including ‘losers’ or other mediocre performers. Such information can greatly facilitate our understanding of and our ability to engineer ribozymes.

Using this method, we analyzed 1024 mutants of the recently discovered twister ribozyme (3) in which five bases involved in or neighboring a pseudoknot interaction (10,11) were randomized. In addition, we assayed the ligand-dependent activities of two aptazyme libraries based on a hepatitis delta virus (HDV)-like ribozyme each of which consisting of 256 variants of the four bases connecting a guanine aptamer and the ribozyme. The ribozyme activities inferred by sequencing showed good correlation with the conventional biochemical assay results of individual mutants. Furthermore, we tested some of the efficient aptazymes identified by sequencing and found that they function as gene switches in mammalian cells when embedded in the 3′ untranslated region (UTR) of a reporter gene. Our strategy described here will facilitate deeper understanding of the sequence–function relationships of natural and engineered ribozymes.

## MATERIALS AND METHODS

### General information

All oligonucleotides were purchased from IDT. The degenerate bases (N) in the oligonucleotides used for library construction were synthesized using IDT's ‘hand-mix’ option (equimolar mix of A, C, G and T). Ethanol precipitation was performed using Quick-Precip Plus Solution (Edge BioSystems).

### Library sequences

The DNA sequences of the libraries used to perform *in vitro* transcription are as follows.

Lib-Tw: 5′TAATACGACTCACTATAGGGCGCGGCATTAATGCAGCTTTATTGGAAACAATAAAGC G**NNNNN**AAGCCCGCAAAAATAGCAGAGTAATGTCGCG3′

Lib-J1/2: 5′TAATACGACTCACTATAGGGCCGCGACTCTAGAAGTGATGCTCTGC**NNNN**ATAATC GCGTGGATATGGCACGCAAGTTTCTACCGGGCACCGTAAATGTCCGACTACTCCTATTCCGGCACGTCCACGTCGTGCAGAGCGGTAACATGCGTTACTAGGGGTGCAAGAGCTCTTTTTGAGGAGGAGCTCTTTTTGCTGCACTAGTTGCATCAGATGGTAACGCATGGCTAAGCCGGAAAGGGGGAGAC3′

Lib-P4: 5′TAATACGACTCACTATAGGGCCGCGACTCTAGAAGTGATGCTCTGCAAATGGGGTA GGAGGCGATGCCTCGTCCTCATACCCAACTCCTATTCCGGCACGTCCACGTCGTGCAGAGCGGTA**NNNN**TATAATCGCGTGGATATGGCACGCAAGTTTCTACCGGGCACCGTAAATGTCCGACTAGTAGCTAAGCCGGAAAGGGGGAGAC3′

(underline: T7 promoter, bold: degenerate bases)

### Construction of HTS libraries

DNA templates encoding a T7 promoter and the ribozyme libraries shown above were prepared using synthetic oligonucleotides and polymerase chain reaction (PCR). The Lib-Tw DNA template was *in vitro* transcribed (37°C, 3 h) in the transcription buffer (40-mM Tris-HCl pH 8.0, 2-mM spermidine, 10-mM DTT) containing T7 RNA polymerase (2.5 U/μl, New England Biolabs), NTPs (2 mM each), MgCl_2_ (4 mM), RiboLock RNase Inhibitor (2 U/μl, Thermo Scientific) and the DNA template (200 nM) in 30-μl volume. For Lib-J1/2 and Lib-P4, 3-mM MgCl_2_ and 300-nM DNA template were used and the reactions were performed in 180-μl scale with or without guanine (500 μM). Upon completion of the transcription reaction of Lib-Tw, 10 U of DNase I (New England Biolabs) in 30 μl (16.6-mM Tris-HCl pH 7.6, 4.2-mM MgCl_2_, 0.83-mM CaCl_2_) was added and incubated on ice for 15 min. For Lib-J1/2 and Lib-P4, 72 U of DNase I in 180 μl (20-mM Tris-HCl pH 7.6, 5-mM MgCl_2_, 1-mM CaCl_2_) was added and incubated on ice for 45 min. RNAs were recovered by ethanol precipitation (Lib-J1/2, Lib-P4) or RNA Clean-up & Concentration kit (Lib-Tw) (Zymo Research) and resuspended in 20–30 μl 0.1-mM ethylenediaminetetraacetic acid (EDTA).

Approximately 60 pmol of the RNAs were mixed with 120 pmol of the reverse transcription primer (Supplementary Table S1) in 17 μl and heated to 95°C for 2 min followed by incubation at 65°C for 5 min and placed on ice. Reverse transcription was initiated by adding 12.3-μl RT buffer (157-mM Tris, pH 8.3, 236-mM KCl, 9.4-mM MgCl_2_, 1.57-mM each dNTPs, 15.7-mM DTT) and 1.5-μl (300 U) SuperScript III Reverse Transcriptase (Life Technologies, 45°C for 1 min, 52°C for 25 min, 65°C for 5 min). The reaction was quenched by addition of 1.5-μl NaOH (5 M) and heat (95°C for 5 min). The cDNAs were purified by denaturing polyacrylamide gel electrophoresis (PAGE) (8% polyacrylamide, 8-M urea, 19:1 acrylamide:bisacrylamide) and recovered in 17 μl 0.1-mM EDTA solution.

The 5′ phosphorylated adapter ADP_B (Supplementary Table S1) was ligated to the cDNAs using Quick Ligation Kit (New England Biolabs) in the presence of appropriate splint oligos (Supplementary Table S1). The ligation reaction contained ∼1-pmol cDNAs, 200 pmol each of splint oligos (for cleaved and uncleaved cDNAs), and 400-pmol ADP_B in 38 μl 1× Quick Ligation Reaction Buffer which was heated to 95°C for 10 min and slowly cooled to 25°C before adding 2-μl Quick T4 DNA Ligase and reacted at 37°C for 6 h. The Lib-Tw ligation was performed at half scale.

The ligated cDNAs were recovered by ethanol precipitation and resuspended in 20 μl of H_2_O. The splint oligos were designed to be complementary to the 5′ end of ADP_B and the 3′ end of the cleaved or uncleaved cDNAs (Supplementary Table S1). To avoid amplification during PCR, mismatched sequences were added to the 3′ ends of the splint oligos. Finally, the sequencing libraries were generated by PCR with primers ADB_T-2 and ADB_B-2 (Supplementary Table S1) using Phusion Flash High-Fidelity PCR Master Mix (Thermo Scientific) in 100-μl volumes containing 5-μl ligation products and 0.5 μM each primer. Nine cycles of 10 s at 98°C followed by 20 s at 72°C were performed and the PCR products were purified by agarose gel electrophoresis using Zymoclean Gel DNA Recovery Kit (Zymo Research).

### Sequencing and data analysis

The libraries were sequenced on an Illumina MiSeq sequencer using MiSeq Reagent Kit v3 (150 cycles, single-end) with 8.1% PhiX control by UC Davis DNA Technologies Core. The reads were first sorted into −guanine and +guanine pools using the 6-base barcode and further sorted according to the sequence identity into the respective libraries. From each sequence read, the identity of the library (Lib-Tw, Lib-J1/2 or Lib-P4), variant (randomized bases) and its status (cleaved or uncleaved) were analyzed and recorded. Finally, the numbers of cleaved and uncleaved reads for each variant were calculated.

### *In vitro* ribozyme assays

Individual ribozyme variants tested were first cloned in a plasmid and sequence verified. The plasmids were used to prepare *in vitro* transcription templates by PCR as described above for the library construction. *In vitro* transcription was performed under the same conditions as the library construction with the exception of the reaction volume (10 μl). The RNAs were separated by 8% (Lib-J1/2 and P4) or 10% (Lib-Tw) denaturing PAGE and stained by SYBR Gold (Life Technologies). The gels were photographed and analyzed using Bio-Rad ChemiDoc MP System. Intensities of the cleaved and uncleaved bands were corrected by the corresponding RNA lengths to estimate the molar fraction of the cleaved ribozyme.

### Aptazyme assay in mammalian cells

Aptazyme sequences were cloned in the 3′ UTR of the EGFP transcript in pEGFP-N1 (Clontech) as previously described (12). HEK293 cells were maintained in a 5% CO_2_ humidified incubator at 37°C in Dulbecco's modified Eagle's medium (Mediatech) supplemented with 10% fetal bovine serum (Gibco) and 1× antibiotic-antimycotic (Gibco). One day before transfection, HEK293 cells were trypsinized and diluted appropriately with fresh complete medium, and 2.4 × 10^4^ cells/well (∼100 μl) were seeded onto 96-well plates. Fifty nanograms of an EGFP -aptazyme plasmid, 50 ng of pL22 (13) (used as an inactive DNA to optimize the transfection condition) and 10 ng of pCMV-mCherry plasmid (constitutively expresses mCherry) were cotransfected using 1 μl of PolyFect reagent (QIAGEN) per well according to the manufacturer's instruction. After 4-h incubation, the medium was removed and replaced with 100-μl fresh complete medium with or without guanine (500 μM). Guanine was first dissolved at 50 mM in 0.2-M NaOH, and was diluted 100-fold with the complete medium immediately before use. The cells were incubated for additional 19 h before EGFP assay.

Cellular fluorescence was measured and normalized according to our previous report (12). Briefly, the cell culture medium was replaced with phosphate buffered saline (PBS) (150 μl per well) and incubated at 37°C until measurement. Fluorescence intensities were measured for EGFP (484-nm excitation/510-nm emission/5-nm bandwidth) and mCherry (587-nm excitation/610-nm emission/10-nm bandwidth) using Safire2 microplate reader (Tecan). The raw fluorescence values were first subtracted with that of the untransfected cells (background). For each well, EGFP fluorescence was normalized by mCherry ([EGFP fluorescence]/[mCherry fluorescence]) to account for variations in transfection efficiency. The values were further normalized by the cells transfected with pEGFP-AgamRz (inactive)/pCMV-mCherry (=1.0). Plasmid pEGFP-AgamRz (inactive) is a control EGFP expression vector in which an inactivated drz-Agam-2-1 was inserted in the same location as the other aptazymes. The reported values are mean ± SD from three replicate samples.

## RESULTS

### Library design and construction

**Figure 2.**
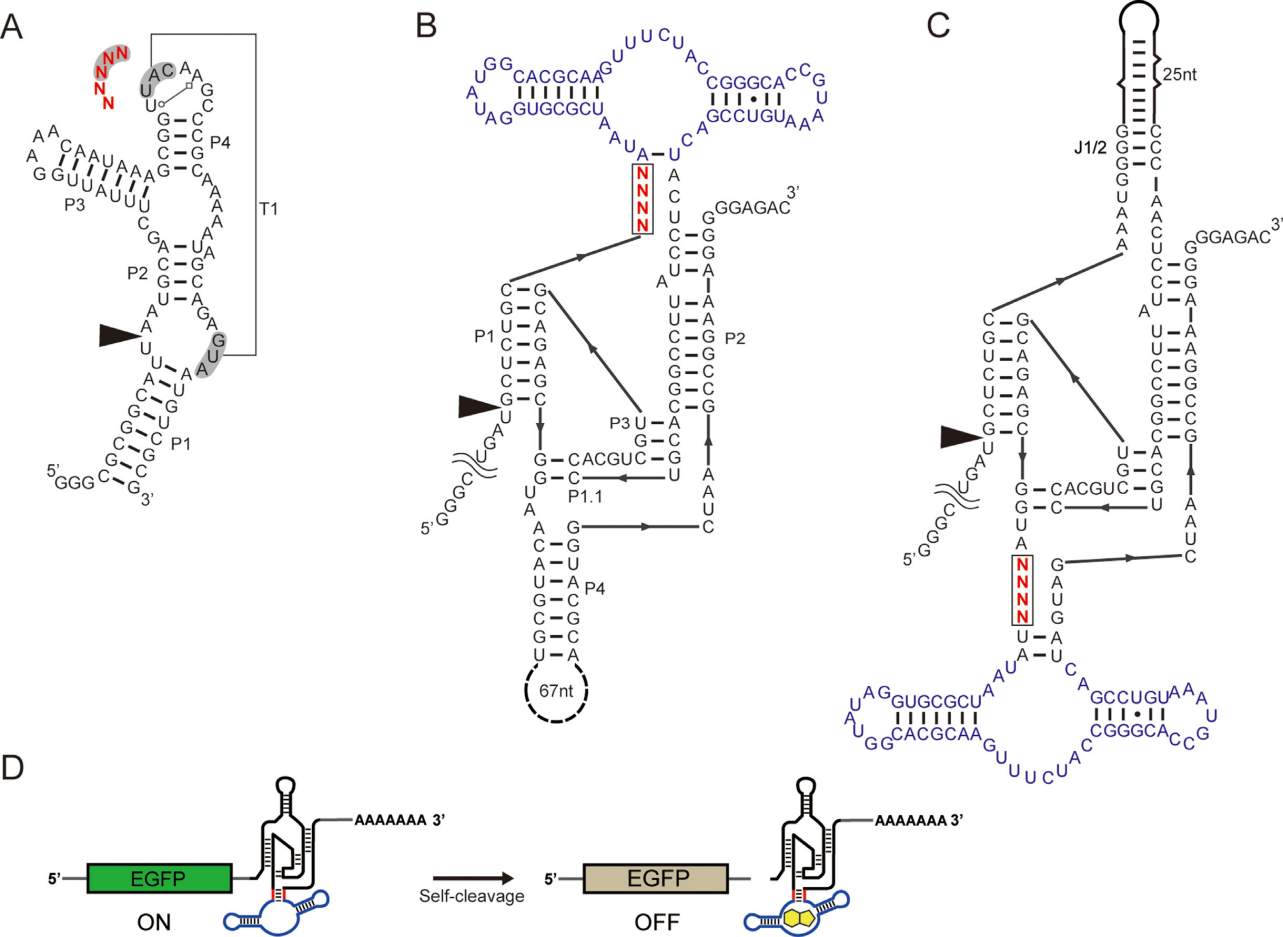
Ribozyme library designs. Degenerate bases are shown in red and the cleavage site is marked by an arrowhead. (**A**) Lib-Tw based on a twister ribozyme discovered from the environmental DNA (3). The bases involved in the T1 pseudoknot are shaded. (**B**) Lib-J1/2 based on the HDV-like ribozyme in *A. gambiae* drz-Agam-2-1(1). The guanine aptamer (purple) was inserted at the J1/2 loop via four random bases. (**C**) Lib-P4 based on drz-Agam-2-1. The P4 stem was replaced with the guanine aptamer (purple) through a randomized connector sequence. (**D**) Illustration of aptazymes used to regulate gene expression in mammalian cells. An aptazyme is embedded in the 3′ UTR of a reporter gene (EGFP) mRNA. Ribozyme cleavage induced by the aptazyme results in low EGFP expression due to the scission of the poly (A) sequence from the coding region.

Three libraries were designed as illustrated in Figure 2. Lib-Tw was based on one of the twister ribozymes recently discovered by the Breaker group (3). Specifically, the ribozyme referred to as ‘environmental sequence’ was modified so that the transcription starts and ends at the P1 helix. Five consecutive bases shown in Figure 2A were randomized. The first two bases (GU) are conserved in >97% of the twister ribozymes and the latter three bases (UAC) engage in a pseudoknot interaction (3,10,11). Lib-J1/2 and Lib-P4 (Figure 2B and C) were based on an HDV-like ribozyme found in *Anopheles gambiae* (drz-Agam-2-1) (1). This particular HDV-like ribozyme contains extensive stem-loop structures in both P4 and J1/2 positions which we speculated may be substituted with an RNA aptamer to engineer an allosteric regulation of the ribozyme activity. The guanine aptamer from a riboswitch in *Bacillus subtilis* (14) was inserted to these positions via four consecutive degenerate bases (Figure 2B and C).

The DNA templates encoding these libraries were synthesized and used to generate the ribozymes by *in vitro* transcription. The RNA library was reverse transcribed using a primer that contains a barcode sequence to identify the reaction conditions (with or without guanine), as well as a portion of the Adapter T sequence used by the Illumina sequencer. After RNA digestion and gel purification, the cDNAs were ligated to the 3′ adapter ADP_B (Supplementary Table S1) using T4 DNA ligase and splint oligos designed for both cleaved and uncleaved DNAs. The ligation efficiencies were optimized and determined to be almost quantitative using control substrates (data not shown). The library was PCR amplified and sequenced using MiSeq Reagent Kit v3 (Illumina).

### Sequencing data processing and analysis

The sequencing results are summarized in Supplementary Table S2. The average number of reads per variant ranged from 1127 to 5873 depending on the library. Importantly, all variants in the libraries had sufficient number of reads to calculate the fraction of the cleaved sequence. For each library, the ribozyme activities (fraction cleaved) of every variant according to the sequencing data were tabulated (Supplementary data set). Reproducibility of the HTS-based ribozyme assay was tested by repeating the library construction and sequencing of Lib-P4 which showed a reasonable agreement (*R*^2^ = 0.90) between the two experiments (Supplementary Figure S1). Some systematic deviations between the two replicates were observed, particularly for the ribozymes with intermediate activities. This may be due in part to the dynamic nature of our samples (ribozymes) which undergo self-cleavage during the initial phase of the library preparation, making some variants more sensitive to small variations in experimental conditions.

### Comparison of sequencing and biochemical data

**Figure 3.**
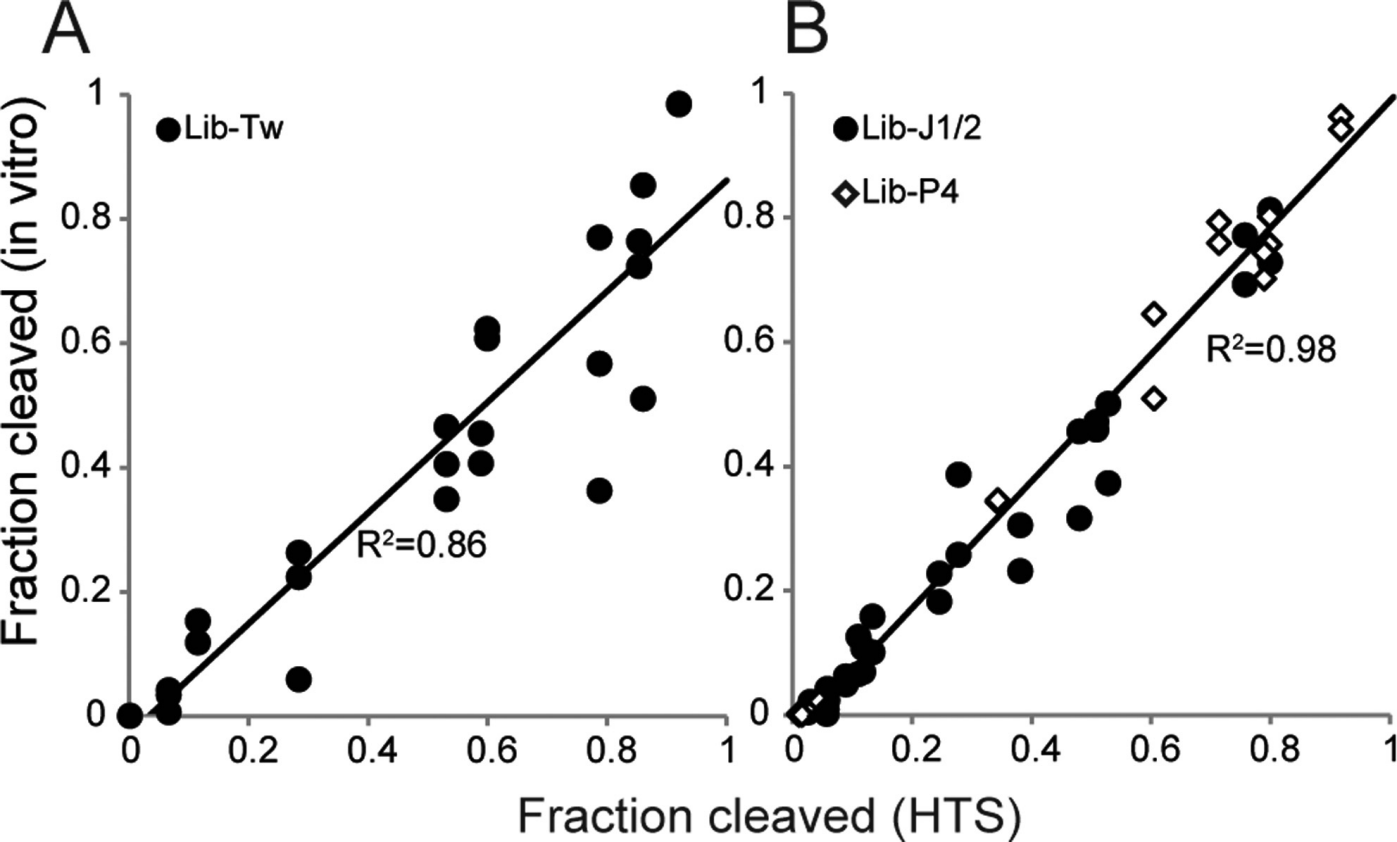
Correlation between the HTS and *in vitro* assays. Ribozyme activities (fraction cleaved) of selected variants were assayed individually by gel electrophoresis (*in vitro*) and plotted against the activities measured by HTS. At least two measurements by gel electrophoresis were performed for each variant. (**A**) Lib-Tw. (**B**) Lib-J1/2 (filled circles) and Lib-P4 (open diamonds).

**Figure 4.**
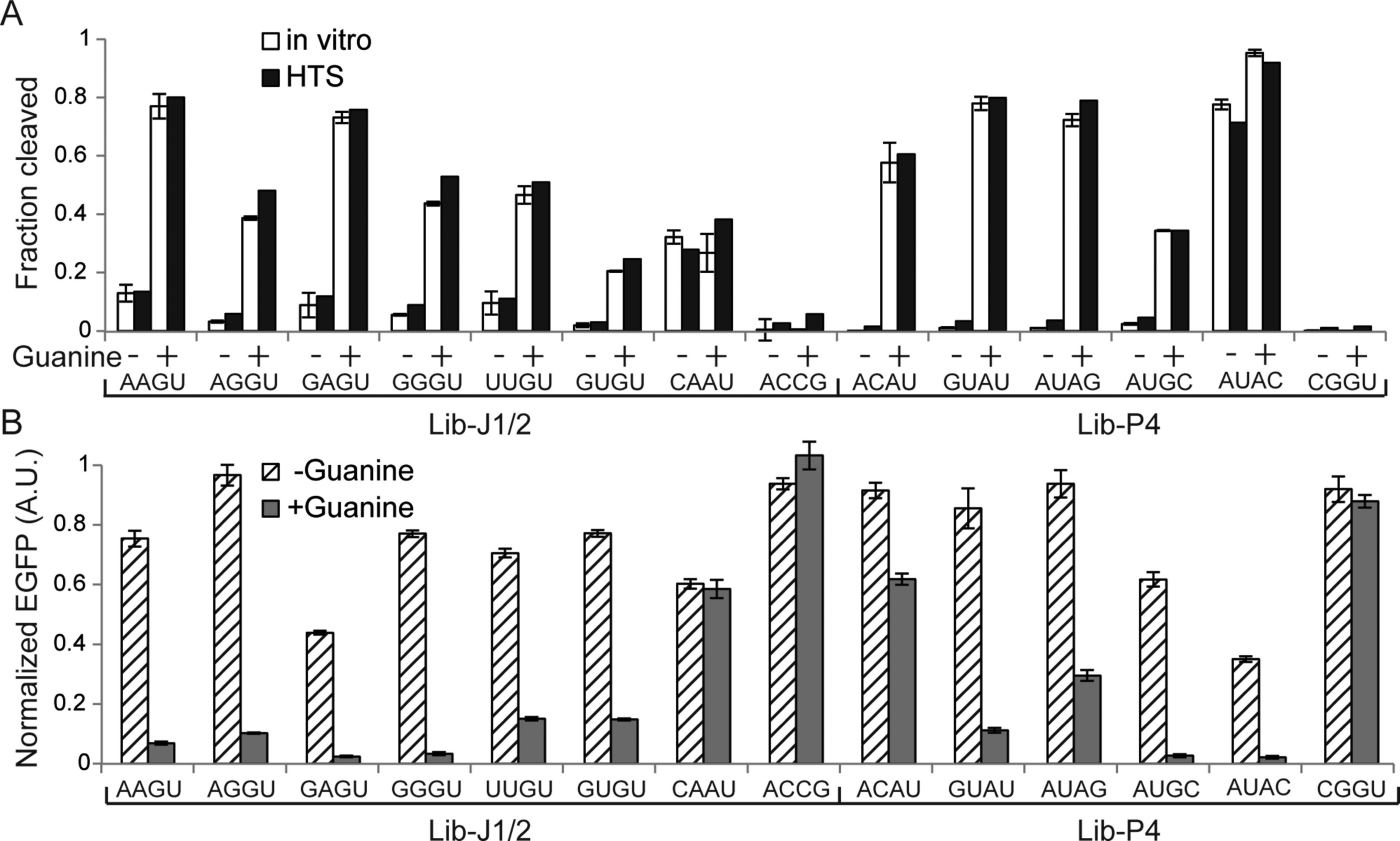
Characterization of the guanine-responsive aptazymes from Lib-J1/2 and Lib-P4. (**A**) Comparison of guanine-dependent ribozyme activities of individual aptazyme variants as measured by gel electrophoresis (*in vitro*, white) and HTS (black). In +guanine reactions, 500-μM guanine was present in the reactions. The error bars (*in vitro*) indicate the ranges of the two independent measurements. (**B**) Expression of EGFP in the HEK 293 cells transfected with EGFP-aptazyme expression plasmids in the absence (hatched) and presence (gray) of guanine. EGFP expression is reported relative to the control which contains an inactivated drz-Agam-2-1 ribozyme (1.0), after normalization for transfection efficiency using the cotransfected mCherry expression plasmid. In +guanine samples, 500-μM guanine was added to the medium. The data shown are mean ± SD from three replicate samples.

After inspecting the sequencing data, we selected several variants from the libraries and assayed their ribozyme activities individually. *In vitro* transcription was performed for each variant using the same conditions used to construct the sequencing library and the products were analyzed by denaturing PAGE. Fraction of the cleaved ribozyme determined by gel electrophoresis was plotted against the results obtained by HTS (Figure 3). The twister variants tested showed a good linear correlation (*R*^2^ = 0.86, slope = 0.89; Figure 3A) while the Lib-J1/2 and Lib-P4 clones exhibited an excellent linear correlation (*R*^2^ = 0.98, slope = 1.00) between the two assays (Figure 3B). While the slopes of the regression line for the twister ribozyme variants slightly smaller than 1.0, it can be attributed to the fact that the HTS assay involves additional biochemical steps (reverse-transcription, adapter ligation and PCR) some of which may contribute to minor systematic biases. For Lib-J1/2 and Lib-P4, guanine responses of the individual ribozymes between the two assays were also consistent (Figure 4A and Supplementary Table S3). Taken together, the ribozyme activities measured by our sequencing method agree with the conventional biochemical assays of individual ribozymes.

### Aptazyme activity in mammalian cells

The selected ribozymes from Lib-J1/2 and Lib-P4 were cloned in the 3′ UTR of the EGFP mRNA as previously described (12) to evaluate their function as chemically controlled gene switches. When the ribozyme is active, separation of the poly (A) sequence from the EGFP coding region results in mRNA degradation and reduced EGFP expression (Figure 2D). The qualitative behaviors of the aptazymes in response to guanine are generally consistent *in vitro* (Figure 4A) and in the HEK 293 cells (Figure 4B) with one exception (Lib-P4:AUAC). The correspondence is remarkable considering the significant differences in the two environments.

## DISCUSSION

The mechanistic studies and engineering of ribozymes frequently involve biochemical characterization of multiple ribozyme mutants. Typically, researchers construct and purify individual mutants and perform assays to quantify ribozyme activity which is highly labor intensive. We demonstrated here that we can obtain equivalent data for >1000 predefined ribozyme variants using HTS. The ribozyme activities (fraction cleaved) of selected Lib-Tw variants determined by HTS were generally in line with the results obtained by gel electrophoresis (Figure 3A). Some variations were observed with some of the partially active twister ribozyme mutants when assayed by gel electrophoresis due to their residual activities during the sample preparation steps (Supplementary Figure S2). When the Lib-Tw library was transcribed, there was no detectable cleavage by PAGE (data not shown). This observation is consistent with the HTS results that indicate there are only nine variants (out of 1024) that exhibit >30% self-cleavage (Table 1 and Supplementary data set). Not surprisingly, the wild-type (Lib-Tw:GUUAC) shows the highest activity and all other partially active ribozymes (fraction cleaved >0.30) are single-base mutants (Table 1 and Supplementary data set).

**Table 1.** Activities of selected ribozyme variants from HTS assay

	Sequence	Fraction cleaved (HTS)	Fraction cleaved (*in vitro*)
	GUUAC^a^	0.92	0.98
	GUUGC	0.86	0.68
	GUUUC	0.85	0.74
	GUUCC	0.80	n.d.
	GUCAC	0.79	0.57
	UUUAC	0.60	0.62
Lib-Tw^b^	GCUAC	0.59	0.43
	AUUAC	0.57	n.d.
	GUUAU	0.53	0.41
	GUAAC	0.28	0.18
	GAUAC	0.12	0.14
	ACUAC	0.07	0.03
	AUCUA	0.0013	0.00^d^
			
	AAGU	0.13/0.80	0.13/0.77
	GAGU	0.12/0.76	0.09/0.73
	UAGU	0.13/0.74	n.d.
Lib-J1/2^c^	CAGU	0.13/0.70	n.d.
	UGGU	0.10/0.61	n.d.
	GGGU	0.09/0.53	0.06/0.44
	UUGU	0.11/0.51	0.10/0.47

Results from *in vitro* PAGE assays are provided where available.

^a^Wild-type sequence.

^b^Deviations from the wild-type sequence are underlined.

^c^−guanine/+guanine. n.d.: not determined.

^d^Cleaved RNA was not visible.

Similarly, the aptazyme libraries Lib-J1/2 and Lib-P4 showed a striking linear correlation between the HTS and biochemical assays (Figures 3B and 4A). Consequently, we can extract some insights into the sequence requirements of the aptazymes within these library constituents from the HTS data. The average activities of Lib-J1/2 variants were 0.092 and 0.169 in the absence and presence of guanine, respectively, indicating that the ribozyme variants generally exhibit low activity (Supplementary data set). When the variants are sorted according to the activity in the presence of guanine, it becomes evident that the stem structure at the base of the aptamer plays a crucial role in modulating the ribozyme activity. The four most active variants are Lib-J1/2:NAGU which are expected to form a 3-bp Watson–Crick stem to support the aptamer folding resulting in 5- to 6-fold activation of ribozyme activity in the presence of guanine (Table 1). Looking at the Lib-P4 data set, Lib-P4:AUAC is the only variant in the library that exhibits strong activity both in the presence (0.92) and absence (0.71) of guanine (Supplementary data set) and it is predicted to form five consecutive Watson–Crick base pairs at the base of the guanine aptamer (Supplementary Figure S3). It is likely that this stem sufficiently stabilizes the ribozyme scaffold even without the guanine-bound aptamer, resulting in the constitutive activity. This hypothesis is consistent with the observation that all single-base mutants of this ribozyme—with a slightly destabilized stem—gain guanine responsiveness (Supplementary Figure S3). Further weakening (i.e. more mismatches) of the stem at this position generally renders the ribozyme less active with or without guanine. It has been observed that although the P4 stem is not absolutely necessary, it appears to enhance the ribozyme activity (15). This kind of analysis where a ribozyme sequence (e.g. from a selection experiment) with a known activity is mutated and assayed to test a mechanistic hypothesis is a common practice when investigating or engineering ribozymes but it is laborious and time-consuming to individually prepare and analyze multiple ribozyme variants. In contrast, our HTS assay provides exhaustive activity data of a given set of ribozyme mutants with which one can test any number of hypotheses within the sequence pool.

HTS ribozyme assay *in vitro* would also be highly useful if at least some results can be extended to *in vivo* activity. Gratifyingly, most of the guanine-responsive aptazymes examined individually (Figure 4A) *in vitro* also functioned as guanine-regulated gene switches when incorporated in the 3′ UTR of a reporter gene mRNA (Figures 2D and 4B). Considering the various parameters that can differentially affect the aptazyme performance *in vitro* and in the cellular environment, the results are of significant interest for biological applications of the engineered ribozymes (8,9). This observation may be due to the fact that we assayed cotranscriptional cleavage activities in a relatively low magnesium ion concentration, conditions which may be more biologically relevant.

**Figure 5.**
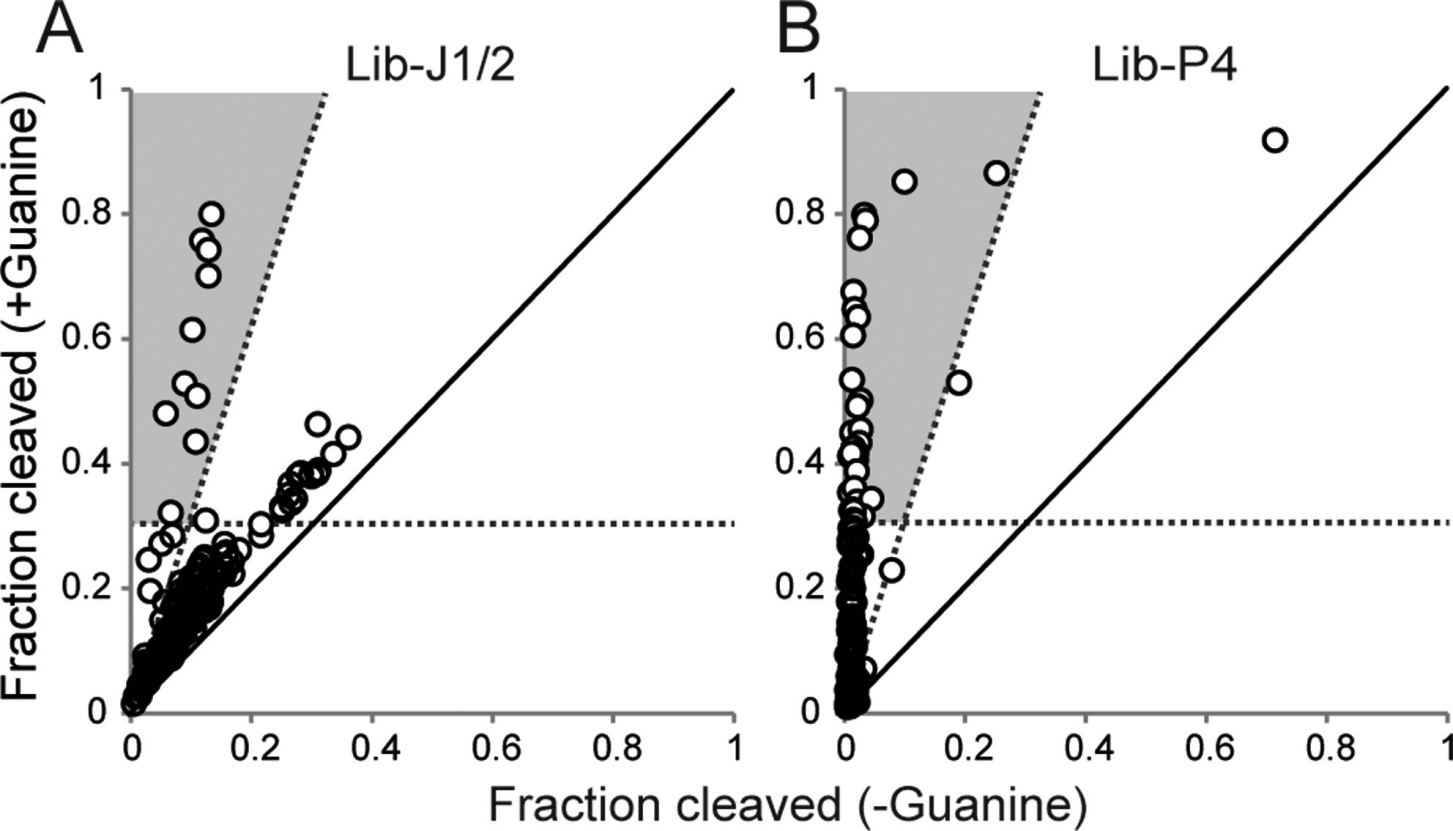
Phenotypic distributions of the aptazyme libraries. Ribozyme activities of the library variants (±guanine) were plotted. The solid line has a slope = 1 which implies no change in ribozyme activity in the presence of guanine. The shaded region represents a guanine-activated aptazyme phenotype that satisfies the following arbitrarily determined criteria: (i) activity in the presence of guanine is >0.30, and (ii) ribozyme is activated at least 3-fold in the presence of guanine (on/off ratio >3). These plots provide a visual overview of the phenotypic distributions of aptazyme libraries. (**A**) Lib-J1/2. (**B**) Lib-P4.

By plotting the activities of all aptazyme variants both in the presence and absence of guanine, the graphs in Figure 5 provide an overview of the phenotypic distributions of the two libraries. The majority of the Lib-J1/2 variants are plotted near the identity line (slope = 1.0) within the fraction cleaved range between 0 and 0.4 (Figure 5A) indicating that they exhibit varying ribozyme activities but do not significantly respond to guanine. However, there are a number of guanine-activated aptazymes that deviate from this trend. On the other hand, vast majority of the Lib-P4 population (252 out 256) is cleaved less than 10% in the absence of guanine but their activities in the presence of guanine range from 0 to 80%. This indicates that the stability of the P4 stem is crucial for the ribozyme activity and that Lib-P4 contains more guanine-activated aptazymes. By setting an arbitrary threshold, ‘functional aptazymes’ in these libraries can be graphically identified as shown in Figure 5. For example, aptazymes that exhibit greater than 3-fold activation in the presence of guanine and show greater than 30% cleavage in the presence of guanine fall within the shaded regions indicated in the two graphs (Figure 5).

Typically, aptazymes are obtained by iterative rational design process, or screening or selection of partially randomized aptazyme libraries (8,16,17). Rational design of aptazymes based on computational prediction of secondary structures does not take into account the true 3D structures of the aptamers and ribozymes and still often requires construction and characterization of many individual aptazyme candidates. For aptazyme applications in the cellular environment, cotranscriptional folding kinetics can also play a role which is often not taken into account in rational design. *In vitro* selection applied to aptazyme design is powerful and can enrich very rare functional aptazymes from complex libraries (16). However, selections are usually carried out in multiple rounds and a large number of clones often need to be screened individually to identify the optimal aptazymes. Therefore, the conventional aptazyme design strategies are laborious and time-consuming.

HTS has been applied to the development and characterization of DNA and RNA aptamers frequently in conjunction with SELEX (18–26). However, applications of HTS to ribozymes have been scarce. Pitt and Ferré-D'Amaré sequenced the populations from a ligase ribozyme selection experiment to infer activities of ∼10^7^ genotypes from the changes in abundance of the individual sequences before and after selection (27). More recently, Ameta *et al*. have used HTS to analyze the progress of *in vitro* selection of ribozymes from random sequences (28). These studies used HTS to track how the ribozyme populations change in the context of *in vitro* selection experiments. While the previous methods are useful for understanding the selection process and dynamics, biochemical information of the large fraction of the unselected sequences, some of which may be due to selection biases (e.g. PCR efficiency), is lost by the selection itself. In addition, sequence data obtained from selection experiments must be analyzed carefully if the objective is to infer the activities of the functional nucleic acids (aptamer affinities, ribozyme activities, etc.) from the abundance of individual sequences in the population. In fact, it has been observed that the abundance of catalytic DNAs in *in vitro* selection populations does not always correlate positively with their catalytic activities (29). This is mainly because the fitness (i.e. abundance) of the individual genotypes in selection experiments is a function of multiple parameters in addition to the activity of the nucleic acid sequence. Our strategy is distinct from other reports that use HTS in combination with selection in that it allows complete, unbiased and quantitative assay of a set of desired ribozyme variants regardless of their activities because it does not involve selection.

Our current protocol has several technical limitations. First, the cleavage site must be located upstream (5′ side) of the randomized bases. This restriction may be circumvented by developing alternative library construction methods such as those developed for small RNA profiling (30). Second, the randomized bases and the cleavage site must be within the number of bases that can be sequenced by HTS. However, this should not be a critical limitation since the presently available sequencing chemistry allows up to 600 bases to be read by paired-end sequencing which is well over the sizes of most natural and synthetic ribozymes.

Although we examined libraries of up to 1024 variants in this study, our strategy should be scalable to examine more complex libraries. For example, assuming 2 × 10^9^ total reads by the currently available HiSeq instrument, one can expect to assay 4 × 10^6^ variants (equivalent to about 11 randomized bases) at an average read number of 500 reads per variant. While selection still remains as the only choice for the discovery of new ribozyme activities from random sequence pools, the complexity of the sequence space that can be accessed by our HTS strategy is more than sufficient for probing and engineering existing ribozyme platforms. Alternatively, the sequencing reads can be divided among multiple libraries using distinct barcodes to assay different ribozymes or reaction conditions in a single sequencing run. In this work, barcodes were used to sequence the three libraries depicted in Figure 2 in a single sequencing session.

Our work broadens the applications of the rapidly evolving sequencing technologies to quantitative assays of catalytic nucleic acids. In this work, we quantified the fraction of cotranscriptionally cleaved ribozymes mimicking the biological environment in which we intended to deploy them as RNA gene regulatory devices. If desired, however, our strategy can be easily modified to acquire kinetic rate constants by constructing multiple sequencing libraries from a single reaction at different time points using appropriate barcodes. It should also be applicable to ribozymes that catalyze other chemical transformations that result in sequence alterations such as ligation and splicing. The increasing capacity of HTS to sequence a large number of bases provides new opportunities to study and/or engineer catalytic nucleotides.

## SUPPLEMENTARY DATA

Supplementary Data are available at NAR Online.

SUPPLEMENTARY DATA
