# Supplementary material for: High-throughput assay and engineering of self-cleaving ribozymes by sequencing
Source: Nucleic Acids Res. 2015 Mar 30;43(13):e85. doi: 10.1093/nar/gkv265 (PMC4513843; doi:10.1093/nar/gkv265)
Supplement: SUPPLEMENTARY DATA [file supp_43_13_e85__index.html]

High-throughput assay and engineering of self-cleaving ribozymes by sequencing — High-throughput assay and engineering of self-cleaving ribozymes by sequencing — SUPPLEMENTARY DATA 

# High-throughput assay and engineering of self-cleaving ribozymes by sequencing

## SUPPLEMENTARY DATA

**Files in this Data Supplement:**

- SUPPLEMENTARY DATA
- SUPPLEMENTARY DATA
