# Supplementary material for: High-throughput assay and engineering of self-cleaving ribozymes by sequencing
Source: Nucleic Acids Res. 2015 Mar 30;43(13):e85. doi: 10.1093/nar/gkv265 (PMC4513843; doi:10.1093/nar/gkv265)
Supplement: SUPPLEMENTARY DATA [file supp_gkv265_nar-00122-met-g-2015-File007.doc]

# Supplementary Information

**High-throughput assay and engineering of self-cleaving ribozymes by sequencing**

Shungo Kobori^1^, Yoko Nomura^1^, Anh Miu^1^ and Yohei Yokobayashi^1,2*^

^1^ Department of Biomedical Engineering, University of California, Davis, Davis, California, 95616 USA

^2^ Nucleic Acid Chemistry and Engineering Unit, Okinawa Institute of Science and Technology Graduate University, Onna, Okinawa, 904 0495, Japan

* To whom correspondence should be addressed. Tel: +1 530 754 9676; Fax: +1 530 754 5739; Email: yoko@ucdavis.edu

**
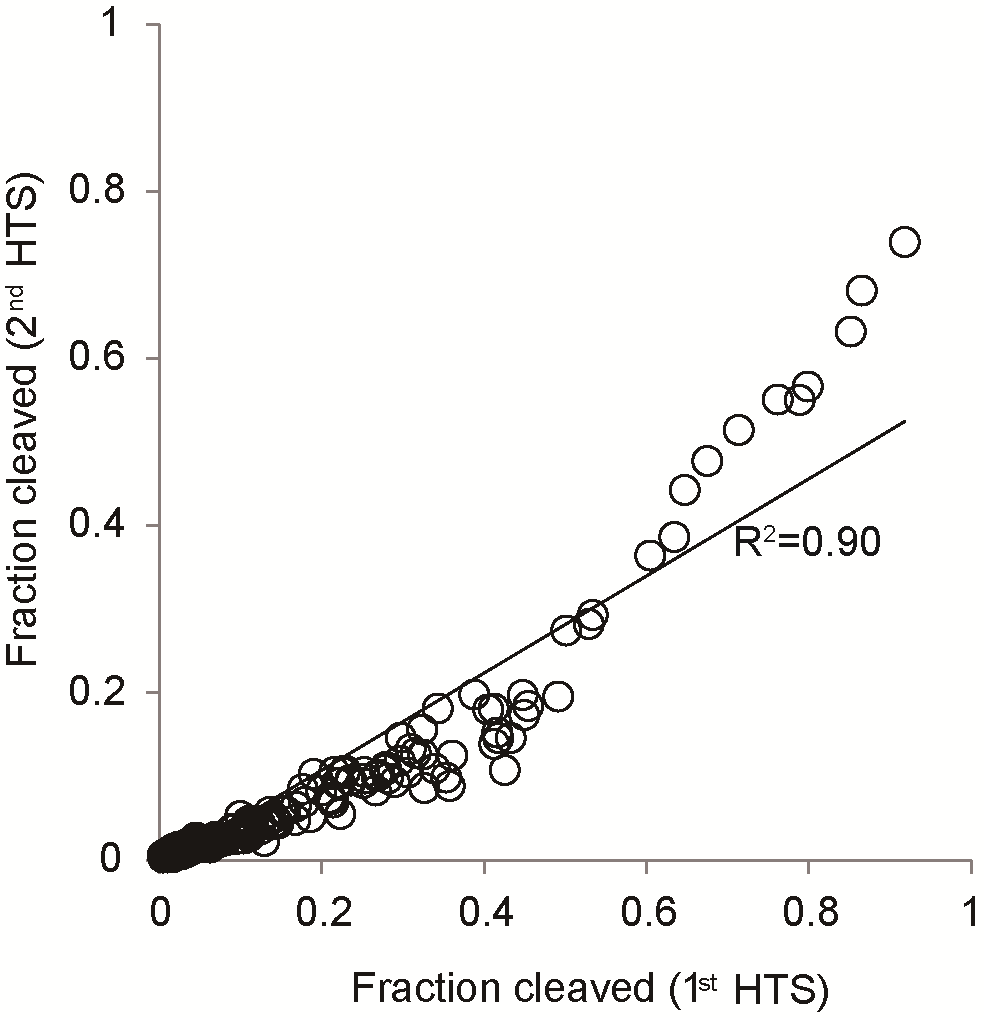
**

**Figure S1.** Reproducibility of HTS assays. Correlation between the two independent preparations and HTS assays of Lib-P4 as described in Material and Methods.

~~.~~


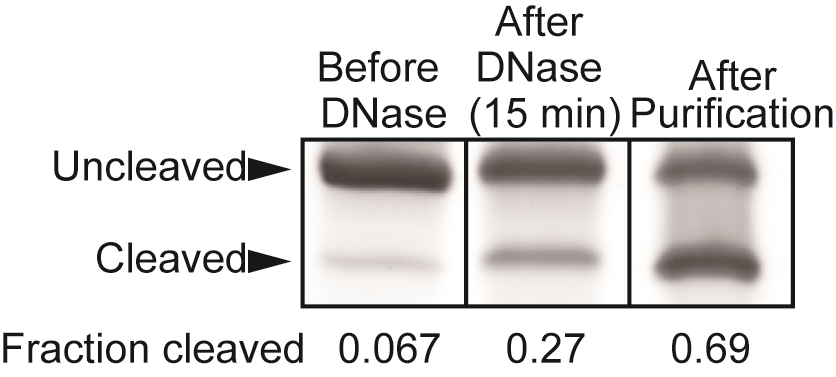


**Figure S2.** A twister ribozyme mutant (Lib-Tw:GUCAC) is active during the sample preparation process before gel electrophoresis. Ribozyme activities were assayed immediately after in vitro transcription, after DNase I reaction (15 min), and after RNA purification and elution in 0.1 mM EDTA. The residual activities of some of the partially active mutants may have contributed to the experimental errors in the gel-based assays.


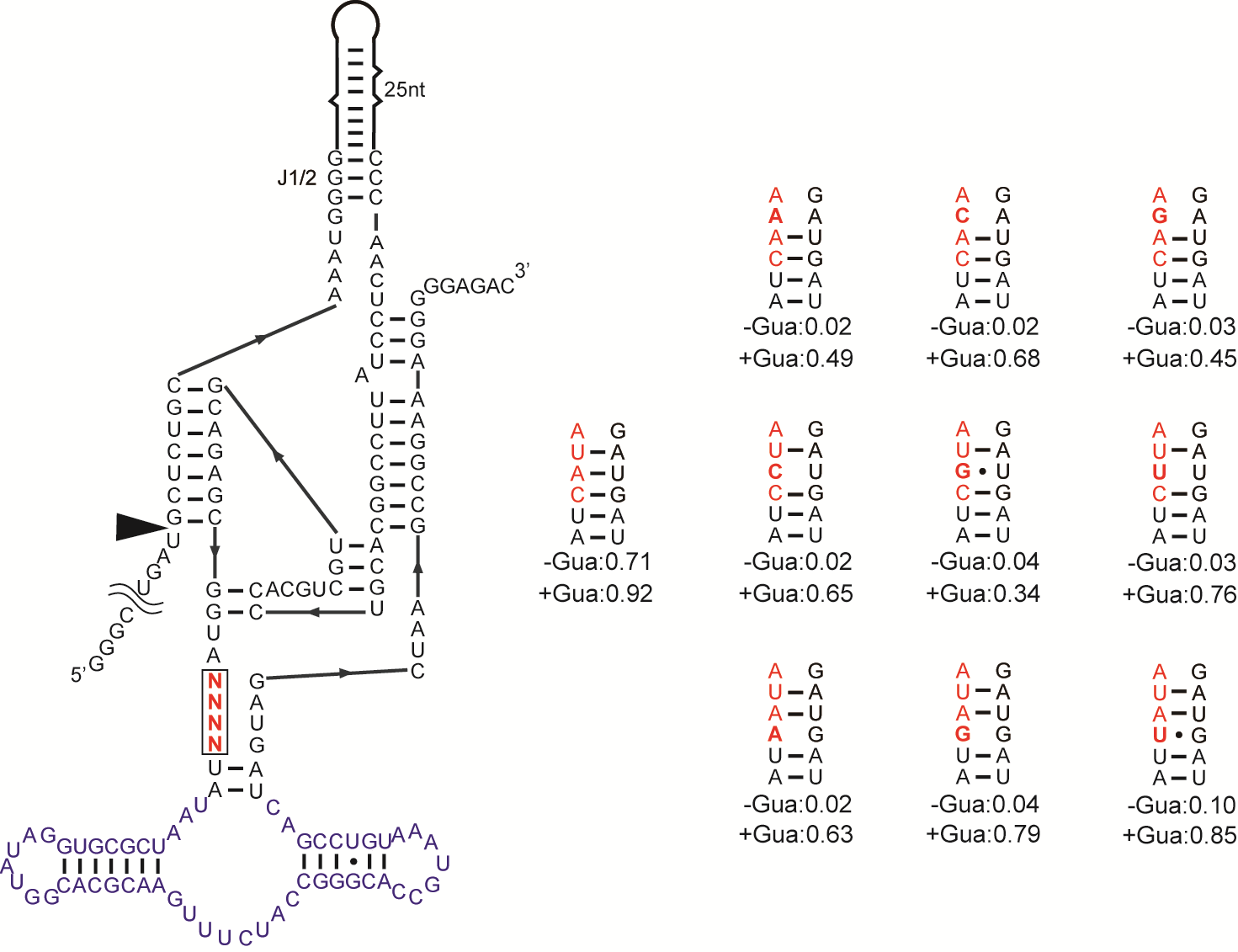


**Figure S3.** Sequence analysis of Lib-P4 variants. Lib-P4:AUAC was found by the HTS assay to be the only variant that exhibit high ribozyme activity in the absence and presence of guanine. Upon examination of the sequence, it was apparent that the variant could form five consecutive Watson-Crick base pairs at the base of the guanine aptamer (boxed region) which may be stable enough to activate the ribozyme in the absence of guanine. The HTS assay allowed us to examine the related variants in the library to further test this hypothesis. Consistent with the hypothesis, all nine single-base mutants of Lib-P4:AUAC exhibit guanine-dependent ribozyme activities. The numbers shown are ribozyme activities (fraction cleaved).

**Table S1.** Oligonucleotides used in this study.

| Name | Sequence (5’ – 3’) | Notes |
| --- | --- | --- |
| RT-Twister | ACACGACGCTCTTCCGATCT**TGCGTT**TACTCTGCTATTTTTGCGGGC | RT primer. Barcode underlined. |
| RT-Guanine- | ACACGACGCTCTTCCGATCT**TGCGTT**TTGCGTGCCATATCCACGCG | RT primer. Barcode underlined. |
| RT-Guanine+ | ACACGACGCTCTTCCGATCT**ACGGCT**TTGCGTGCCATATCCACGCG | RT primer. Barcode underlined. |
| ADP_B | P-AGATCGGAAGAGCGGTTCAGCAGGAATGCCGAGACC-C3 | 3’ Adapter: 5’-phosphorylated, 3’-OCH_2_CH_2_CH_2_OH (C3 spacer) modified. |
| Sp-Tw-cleaved | CCGCTCTTCCGATCTAATGCTCGA | Splint oligo for cleaved Lib-Tw cDNA. |
| Sp-Tw-uncleaved | CCGCTCTTCCGATCTGGGCGGCCG | Splint oligo for uncleaved Lib-Tw cDNA. |
| Sp-Apt-cleaved | CCGCTCTTCCGATCTGCTCTCGTT | Splint oligo for cleaved Lib-J1/2 and Lib-P4 cDNAs. |
| Sp-Apt-uncleaved | CCGCTCTTCCGATCTGGGCCCGCT | Splint oligo for uncleaved Lib-J1/2 and Lib-P4 cDNAs. |
| ADB_T-2 | AATGATACGGCGACCACCGAGATCTACACTCTTTCCCTACACGACGCTCTTCCGATCT | Final PCR primer. |
| ADB_B-2 | CAAGCAGAAGACGGCATACGAGATCGGTCTCGGCATTCCTGCTGA | Final PCR primer. |

**Table S2. Summary of HTS statistics.**

|  |  |  |  | Monomer ratio in the randomized region | | | |
| --- | --- | --- | --- | --- | --- | --- | --- |
| Library | Reads | Average reads per variant | S.D. | A | T | G | C |
| Lib-J1/2 -Guanine | 1,451,836 | 5,671 | 1,037 | 27.8% | 24.1% | 22.9% | 25.3% |
| Lib-J1/2 +Guanine | 1,503,535 | 5,873 | 1,182 | 28.0% | 24.1% | 22.6% | 25.3% |
| Lib-P4 -Guanine | 756,231 | 2,770* | 682* | 28.4% | 24.2% | 24.8% | 22.6% |
| Lib-P4 +Guanine | 891,714 | 3,273* | 831* | 28.6% | 24.3% | 24.7% | 22.5% |
| Lib-Tw | 1,154,124 | 1,127 | 310 | 23.6% | 27.2% | 25.2% | 24.1% |

*The variant AUGC was excluded from calculation because this sequence was used as a template for the PCR during library construction and was overrepresented in the libraries (see Supplementary Data).

**Table S3. Summary of selected ribozyme activities measured by HTS and PAGE.**

|  |  | -Guanine | | | | +Guanine | | | |
| --- | --- | --- | --- | --- | --- | --- | --- | --- | --- |
|  |  | HTS | | | PAGE* | HTS | | | PAGE* |
|  | Sequence | Uncleaved reads | Cleaved reads | Fraction cleaved | Fraction cleaved | Uncleaved reads | Cleaved reads | Fraction cleaved | Fraction cleaved |
| Lib-J1/2 | AAGU | 6085 | 943 | 0.13 | 0.13 | 1560 | 6232 | 0.80 | 0.77 |
|  | AGGU | 5723 | 355 | 0.058 | 0.032 | 3675 | 3398 | 0.48 | 0.39 |
|  | GAGU | 4913 | 661 | 0.12 | 0.088 | 1517 | 4735 | 0.76 | 0.73 |
|  | GGGU | 6331 | 619 | 0.089 | 0.055 | 3620 | 4055 | 0.53 | 0.44 |
|  | UUGU | 4933 | 612 | 0.11 | 0.10 | 2916 | 3020 | 0.51 | 0.47 |
|  | GUGU | 5595 | 172 | 0.030 | 0.019 | 4346 | 1418 | 0.25 | 0.20 |
|  | CAAU | 4217 | 1626 | 0.28 | 0.32 | 4044 | 2492 | 0.38 | 0.27 |
|  | ACCG | 6012 | 160 | 0.026 | 0.00^a^ | 5577 | 340 | 0.057 | 0.00^a^ |
| Lib-P4 | ACAU | 3668 | 55 | 0.015 | 0.00^a^ | 1902 | 2917 | 0.61 | 0.58 |
|  | GUAU | 2444 | 84 | 0.033 | 0.011 | 748 | 2972 | 0.80 | 0.78 |
|  | AUAG | 2870 | 106 | 0.036 | 0.010 | 997 | 3738 | 0.79 | 0.72 |
|  | AUGC | 47521 | 2238 | 0.045 | 0.024 | 37476 | 19599 | 0.34 | 0.34 |
|  | AUAC | 867 | 2163 | 0.714 | 0.78 | 417 | 4694 | 0.92 | 0.95 |
|  | CGGU | 2163 | 24 | 0.011 | 0.00^a^ | 2712 | 43 | 0.016 | 0.00^a^ |
| Twister | GUUAC  GUUGC  GUUUC  GUCAC  UUUAC  GCUAC  GUUAU  GUAAC  GAUAC | 53  106  101  119  374  242  350  768  1046 | 614  654  591  442  561  347  397  305  136 | 0.92  0.86  0.85  0.79  0.60  0.59  0.53  0.28  0.12 | 0.98  0.68  0.74  0.57  0.62  0.43  0.41  0.18  0.14 |  |  |  |  |
|  | ACUAC  AUCUA | 1106  797 | 79  1 | 0.07  0.0013 | 0.03  0.00^a^ |  |  |  |  |

*The values are averages of two independent measurements. ^a^ Cleaved RNA was not visible.
